# Using clinical information to make individualized prognostic predictions in people at ultra high risk for psychosis

**DOI:** 10.1016/j.schres.2016.11.047

**Published:** 2017-06

**Authors:** Andrea Mechelli, Ashleigh Lin, Stephen Wood, Patrick McGorry, Paul Amminger, Stefania Tognin, Philip McGuire, Jonathan Young, Barnaby Nelson, Alison Yung

**Affiliations:** a Department of Psychosis Studies, Institute of Psychiatry, Psychology & Neuroscience, King's College London, London, UK; b Telethon Kids Institute, University of Western Australia, Subiaco, Western Australia 6008, Australia; c Department of Psychology, University of Birmingham, Birmingham, UK; d Melbourne Neuropsychiatry Centre, Department of Psychiatry, University of Melbourne & Melbourne Health, Melbourne, Australia; e Orygen, The National Centre of Excellence in Youth Mental Health, Melbourne, Australia; f The Centre for Youth Mental Health, The University of Melbourne, Melbourne, Australia; g Department of Neuroimaging, Institute of Psychiatry, Psychology & Neuroscience, King's College London, London, UK; h Institute of Brain, Behaviour, and Mental Health, University of Manchester, Manchester, UK; i Greater Manchester West NHS Mental Health Foundation Trust, Prestwich, Manchester, UK

**Keywords:** Ultra-high risk, Psychosis, Clinical outcome, Functional outcome, Support vector machine

## Abstract

Recent studies have reported an association between psychopathology and subsequent clinical and functional outcomes in people at ultra-high risk (UHR) for psychosis. This has led to the suggestion that psychopathological information could be used to make prognostic predictions in this population. However, because the current literature is based on inferences at group level, the translational value of the findings for everyday clinical practice is unclear. Here we examined whether psychopathological information could be used to make individualized predictions about clinical and functional outcomes in people at UHR. Participants included 416 people at UHR followed prospectively at the Personal Assessment and Crisis Evaluation (PACE) Clinic in Melbourne, Australia. The data were analysed using Support Vector Machine (SVM), a supervised machine learning technique that allows inferences at the individual level. SVM predicted transition to psychosis with a specificity of 60.6%, a sensitivity of 68.6% and an accuracy of 64.6% (*p* < 0.001). In addition, SVM predicted functioning with a specificity of 62.5%, a sensitivity of 62.5% and an accuracy of 62.5% (*p* = 0.008). Prediction of transition was driven by disorder of thought content, attenuated positive symptoms and functioning, whereas functioning was best predicted by attention disturbances, anhedonia–asociality and disorder of thought content. These results indicate that psychopathological information allows individualized prognostic predictions with statistically significant accuracy. However, this level of accuracy may not be sufficient for clinical translation in real-world clinical practice. Accuracy might be improved by combining psychopathological information with other types of data using a multivariate machine learning framework.

## 1 Introduction

The onset of a psychotic disorder is typically preceded by a prodromal phase, known as the ultra high risk (UHR) state, involving the emergence of attenuated positive symptoms and a marked decline in functioning (Fusar-Poli et al., 2013, Yung et al., 1996). With the increasing appreciation of the clinical benefits of early intervention in psychosis (McGorry et al., 2008), a number of pharmacological and psychological treatments are being employed to delay or prevent the onset of the illness in people at UHR (Mechelli et al., 2015). Because approximately two-thirds of people who meet criteria for UHR will *not* develop the disorder, treatment that is intended to be preventative may be provided to individuals who may not actually need it. Therefore, the development of predictive tools, that could be used to tailor clinical intervention to the level of risk amongst people at UHR, has become a major translational goal for psychiatric research (Nelson and Yung, 2010).

An association between psychopathology and subsequent clinical outcome in people at UHR for psychosis has been found in a number of studies. The most consistent finding is a positive correlation between severity of bizarre thinking/unusual thought content and risk of transition to psychosis which has been observed in four independent samples (Cannon et al., 2008, Thompson et al., 2011, Velthorst et al., 2009, Ziermans et al., 2014). Other aspects of psychopathology found to be predictive of transition to psychosis in this population include the presence of brief limited intermitted psychotic symptoms (Nelson et al., 2011), severity of positive symptoms (Ziermans et al., 2014), elevated mood (Thompson et al., 2013), severity of delusions (Thompson et al., 2013), basic self-disturbance (Nelson et al., 2012) and disorganised communication (Addington et al., 2015). In addition, disorganised symptoms (Carrion et al., 2013, Ziermans et al., 2014) and negative symptoms (Lin et al., 2011, Meyer et al., 2014) have been found to be predictive of functional outcomes irrespective of transition to psychosis. Collectively, these findings indicate that it may be possible to use careful clinical assessment to predict transition to psychosis as well as psychosocial functioning in individuals at UHR for psychosis.

A critical limitation of the above literature, however, is that the studies published so far typically reported effects that were statistically significant at the group level, whereas clinicians have to make treatment decisions about individual patients. Because effects that are statistically significant at a group level do not necessarily permit accurate inferences at the level of the individual, the translational potential of the findings for everyday clinical practice is unclear. One way of addressing this limitation is to employ supervised machine learning techniques, such as support vector machine (SVM), which permit statistical inferences at the level of the individual and as such have high translational potential in clinical practice (Orru et al., 2012).

While several studies have applied supervised machine learning techniques to neuroimaging and neurocognitive data to predict clinical and functional outcomes in people at UHR for psychosis (Kim et al., 2011, Koutsouleris et al., 2012a, Koutsouleris et al., 2012b, Koutsouleris et al., 2009, Simon et al., 2012, Tognin et al., 2013), to our knowledge no previous investigation has employed this approach to examine the prognostic value of clinical information. The aim of the present study was therefore to examine whether clinical information acquired at baseline could be used to make individualized predictions about long-term clinical and functional outcomes in people at UHR for psychosis. We used longitudinal data from service users at the Personal Assessment and Crisis Evaluation (PACE) clinic, Orygen Youth Health. Participants received a detailed psychopathological assessment at first clinical presentation and were followed-up at regular intervals for an average period of 7.5 years; full details of the protocol can be found in Nelson et al. (2013) (Nelson et al., 2013). Based on the existing literature that used group-level statistics (Cannon et al., 2008, Carrion et al., 2013, Meyer et al., 2014, Nelson et al., 2013, Nelson et al., 2011, Thompson et al., 2013, Thompson et al., 2011, Velthorst et al., 2009, Ziermans et al., 2014), we tested two related hypotheses. First, psychopathological measures including a combination of positive and negative symptoms and functioning variables would allow individualized prediction of transition to psychosis with statistically significant accuracy; more specifically, we expected prediction to be driven by the presence of disorder of thought content, intensity of attenuated positive symptoms and poor functioning (Cannon et al., 2008, Nelson et al., 2013, Thompson et al., 2011, Velthorst et al., 2009, Ziermans et al., 2014). Second, psychopathological measures would also allow individualized prediction of functional outcome with statistically significant accuracy; in this case we expected prediction to be mainly informed by disorganised (Carrion et al., 2013, Ziermans et al., 2014) and negative (Meyer et al., 2014, Nelson et al., 2013) symptoms.

## 2 Materials and methods

### 2.1 Setting and sample

The PACE clinic is a specialist clinic for people at UHR for psychosis. The catchment area of the service includes northwestern metropolitan Melbourne, Australia. Young people between the age of 15 and 30 are accepted into PACE if they meet criteria for at least one of three UHR groups: (i) attenuated psychotic symptoms (APS), (ii) brief limited intermitted psychotic symptoms (BLIPS), and (iii) trait risk factor (Trait) (Yung et al., 2003). Exclusion criteria for the PACE clinic are the presence of a current or past psychotic disorder, known organic cause for presentation, and past neuroleptic exposure equivalent to a total continuous haloperidol dose of > 15 mg (which may modify risk of transition).

A total of 416 people (200 males, 216 female) who met criteria for UHR for psychosis were included in the present investigation (mean age = 19.38, SD = 3.35). All were recruited between 1993 and 2006 and followed up for an average of 7.5 years (median: 8.04, range: 2.4–14.9). Within the sample, 114 individuals (27%) had made transition to psychosis during the follow-up period whereas the remaining 302 (73%) had not. The demographic and clinical characteristics of this sample have been reported and discussed in detail in a previous publication (Nelson et al., 2013). The study was approved by the local ethics committee and written informed consent was obtained from all participants.

### 2.2 Baseline measures

**Fig. 1:**
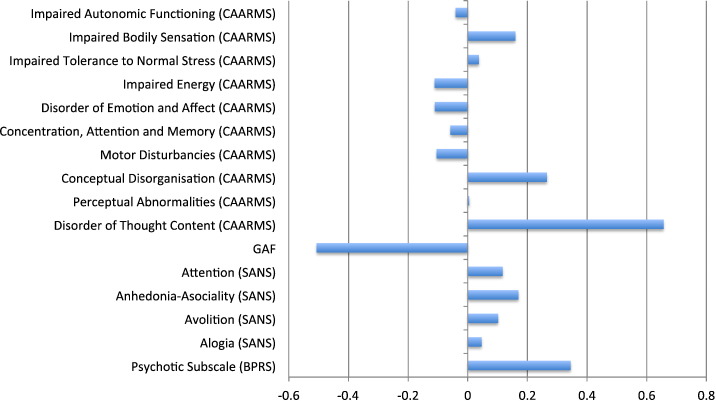
Relative contributions of clinical measures to prediction of long-term clinical outcome (i.e. converters versus non-converters). A positive weight value indicates that a measure contains valuable information for identifying converters, whereas a negative value indicates that it contains valuable information for identifying non-converters.

A range of clinical measures acquired at baseline were used to predict clinical and functional outcomes including the Brief Psychiatric Rating Scale (BPRS); the Scale for Assessment of Negative Symptoms, (SANS); the Comprehensive Assessment of At Risk Mental State (Yung et al., 2005) (CAARMS); and the Global Assessment of Functioning (GAF). See Fig. 1 and Supplementary data for list of specific subscales.

### 2.3 Outcome measures

The main outcome measure of interest was transition to psychotic disorder. This was defined as at least one fully positive psychotic symptom several times a week for more than one week using both the BPRS and the CAARMS (Yung et al., 2004). A further outcome measure of interest was level of functioning at last follow-up. This was assessed using the Social and Occupational Functioning Assessment Scale (SOFAS), with a follow-up score > 50 indicating good functioning and a follow-up score ≤ 50 indicating poor functioning; this cut-off was chosen as it is often used to distinguish between poor and good functioning in clinical practice.

### 2.4 Support vector machine

The data were analysed using SVM as implemented in PROBID software (http://www.kcl.ac.uk/ioppn/depts/neuroimaging/research/imaginganalysis/Software/PROBID.aspx). SVM is a multivariate machine learning technique that allows the classification of individual observations into distinct groups using the rules of probability (see Supplementary Data for more detail) (Vapnik, 1999). SVM comprises a “training” phase, in which well characterized training data are used to develop an algorithm which captures the key differences between groups, and a “testing” phase, in which the algorithm is used to predict the group that a new observation belongs to (Orru et al., 2012). For the purpose of the present investigation, a predictive algorithm was developed using a radial basis function kernel and leave-one-out cross-validation. This involved: (i) excluding a single subject from each group; (ii) training the classifier using the remaining subjects; (iii) using the subject pair excluded to test the ability of the classifier to reliably distinguish between groups; and (iv) repeating this procedure for each subject pair in order to assess the generalizability of the classifier in terms of accuracy, sensitivity and specificity. The statistical significance of the accuracy was determined by permutation testing; this involved repeating the classification procedure with a different random permutation of the training group labels 1000 times, and dividing the number of permutations achieving higher sensitivity and specificity than the true labels by the total number of permutations.

Two distinct SVM analyses were carried out to address the following questions. Firstly, can clinical data predict subsequent transition to psychosis at the individual level (i.e. transition versus non-transition)? Secondly, can clinical data predict subsequent level of functioning at the individual level (i.e. poor versus good functioning)? Ideally, the use of SVM to compare two groups of interest requires them to have the same sample size; furthermore, to maximize the external validity of the findings, participants in the two groups should be matched on basic demographic variables, i.e. age and gender (Orru et al., 2012). In order to address the first question, we therefore selected 99 transitioned participants and 99 non-transitioned participants individually matched for gender and age (± 2 years); the demographic and clinical characteristics of these two groups are reported in Table 1. In order to address the second question, we selected 48 participants with a follow-up SOFAS score > 50, who were classified as high functioning, and 48 participants with a follow-up SOFAS score ≤ 50, who were classified as low functioning, individually matched for gender and age (± 2 years); the demographic and clinical characteristics of these groups are also reported in Table 1. In addition, because functioning can also be thought of as a continuous variable, we carried out a further machine learning analysis of high- and low- functioning participants using an alternative version of SVM known as Support Vector Regression (SVR) (Smola and Scholkopf, 2004). The advantage of SVR, relative to SVM, is that it allows the quantitative prediction of a variable of interest (e.g. a patient's score on a scale of interest) without the need for a discrete categorical decision (e.g. low vs. high functioning). SVR was implemented in Scikit-learn (http://scikit-learn.org/stable/) using a radial basis function kernel and a nested cross-validation design. The inner 10-fold loop optimised the values of the parameters to be estimated (C, epsilon and gamma), while the outer 10-fold loop tested these parameters in subjects not used in training. This was repeated 100 times with random shuffling of the data to produce a variety of different train/test splits, and all the final sets of predictions were then averaged. The statistical significance of this final set of prediction was estimated using a permutation test whereby the actual and predicted scores were randomly paired 1000 times and a new SVR was run for each random pairing. Statistical inferences were made at *p* < 0.05 with Bonferroni correction for multiple comparisons to account for the number of outcome measures investigate (i.e. transition and functioning); this resulted in an actual *p*-value of *p* < 0.025 for each statistical comparison.

**Table 1:** Demographic and clinical characteristics of participants. The participants used for the analysis of functioning were a subset of the participants used for the analysis of transition. Values denote mean with standard error in brackets. *n* = number of subjects in each group; UHR-T = individuals at ultra-high risk who made transition to psychosis; UHR-T = individuals at ultra-high risk who did not make transition to psychosis; Poor = individuals who showed a SOFAS score ≤ 50 at follow-up indicating poor functioning; Good = individuals who showed a SOFAS score > 50 at follow-up indicating good functioning. The asterisk (*) indicates that this information was available for 75/98 individuals who made transition and 77/98 who did not make transition to psychosis.

	Prediction of transition			Prediction of functioning		
	UHR-T (*n* = 99)	UHR-NT (*n* = 99)	Comparison	Low (*n* = 48)	High (*n* = 48)	Comparison
Age	19.52 (3.62)	19.40 (3.37)	*t* = 0.223 df = 196 *p* = 0.824	19.71 (3.06)	19.65 (3.62)	*t* = 0.091 df = 94 *p* = 0.928
Gender (male/female)	48/51	48/51	*×*^2^ < 0.001 df = 1 *p* = 1	22/26	22/26	*×*^2^ < 0.001 df = 1 *p* = 1
Time between baseline assessment and follow-up (days)	3203.19 (1020.341)	2965.79 (1194.932)	*t* = 1.316^⁎^ df = 150 *p* = 0.190	2833.44 (1262.114)	2630.33 (1061.003)	*t* = 0.853 df = 94 *p* = 0.396
Psychotic subscale (BPRS)	10.11 (3.2)	8.63 (2.67)	*t* = 3.54 df = 196 *p* = 0.001	10.29 (3.3)	9.69 (2.86)	*t* = 0.959 df = 94 *p* = 0.340
Alogia (SANS)	2.57 (2.85)	1.99 (2.38)	*t* = 1.54 df = 196 *p* = 0.125	3.44 (3.16)	2.13 (1.86)	*t* = 2.474 df = 94 *p* = 0.015
Avolition (SANS)	4.57 (3.36)	3.65 (2.88)	*t* = 2.065 df = 196 *p* = 0.040	4.92 (3.29)	3.90 (3.18)	*t* = 1.544 df = 94 *p* = 0.126
Anhedonia–Asociality (SANS)	6.78 (4.92)	5.44 (4.65)	*t* = 1.956 df = 196 *p* = 0.052	7.65 (5.15)	5.19 (3.78)	*t* = 2.662 df = 94 *p* = 0.009
Attention (SANS)	2.03 (2.17)	1.28 (1.8)	*t* = 2.631 df = 196 *p* = 0.090	2.48 (2.35)	0.96 (1.28)	*t* = 3.930 df = 94 *p* < 0.001
GAF	54.54 (11.31)	61.10 (11.81)	*t* = − 3995 df = 196 *p* < 0.001	55.27 (11.2)	58.96 (9.51)	*t* = − 1.738 df = 94 *p* = 0.085
Disorder of thought content (CAARMS)	2.29 (0.95)	1.66 (1.05)	*t* = 4.468 df = 196 *p* < 0.001	2.19 (0.86)	1.85 (1.03)	*t* = 1.714 df = 94 *p* = 0.091
Perceptual abnormalities (CAARMS)	2.45 (1.45)	2.11 (1.44)	*t* = 1.662 df = 196 *p* = 0.098	2.42 (1.36)	2.40 (1.55)	*t* = 0.070 df = 94 *p* = 0.945
Conceptual disorganisation (CAARMS)	2.04 (1.02)	1.73 (1.15)	*t* = 2.019 df = 196 *p* = 0.045	2.00 (1.05)	1.69 (1.05)	*t* = 1.453 df = 94 *p* = 0.149
Motor disturbances (CAARMS)	0.75 (1.06)	0.61 (1.01)	*t* = 0.956 df = 196 *p* = 0.340	0.69 (1.03)	0.67 (1.07)	*t* = 0.097 df = 94 *p* = 0.923
Disorder of concentration, attention and memory (CAARMS)	2.18 (1.19)	1.92 (1.14)	*t* = 1.58 df = 196 *p* = 0.116	2.21 (1.23)	2.23 (0.973)	*t* = − 0.092 df = 94 *p* = 0.927
Disorder of emotion and affect (CAARMS)	1.82 (1.32)	1.54 (1.28)	*t* = 1.521 df = 196 *p* = 0.130	2.04 (1.32)	1.83 (1.13)	*t* = 0.829 df = 94 *p* = 0.409
Impaired energy (CAARMS)	1.92 (1.18)	1.90 (1.12)	*t* = 0.123 df = 196 *p* = 0.902	1.83 (1.13)	1.97 (1.02)	*t* = − 0.662 df = 94 *p* = 0.510
Impaired tolerance to normal stress (CAARMS)	1.95 (1.25)	1.79 (1.18)	*t* = 0.93 df = 196 *p* = 0.354	1.90 (1.20)	2.00 (1.07)	*t* = − 0.447 df = 94 *p* = 0.656
Impaired bodily sensation (CAARMS)	0.96 (1.25)	0.70 (1.06)	*t* = 1.59 df = 196 *p* = 0.113	0.60 (1.10)	1.04 (1.12)	*t* = − 1.918 df = 94 *p* = 0.058
Impaired autonomic functioning (CAARMS)	1.04 (1.25)	0.84 (1.13)	*t* = 1.191 df = 196 *p* = 0.235	1.21 (1.23)	1.21 (1.23)	*t* < 0.001 df = 94 *p* = 1.000

## 3 Results

### 3.1 Prediction of transition to psychosis

SVM was able to discriminate between individuals at UHR who subsequently did and did not make transition to psychosis with specificity of 60.6%, a sensitivity of 68.6% and an accuracy of 64.6%; permutation testing indicated that this was statistically significant (*p* < 0.001).

The relative contributions of the different symptoms to prediction of transition to psychosis are displayed in Fig. 1. Here a positive weight means that the measure in question contained valuable information for identifying individuals who made transition, whereas a negative weight means that it was useful for identifying individuals who did not make transition. Individualized predictions were mainly driven by three measures. The first of these measures was disorder of thought content as indexed by the CAARMS, which was higher in the transition than the non-transition group (*p* < 0.001; see Table 1) and added to the prediction of those individuals who made transition. The second measure was intensity of attenuated positive symptoms as indexed by the psychotic subscale of the BPRS, which was higher in the transition than the non-transition group (*p* = 0.001; see Table 1) and added to the prediction of those individuals who made transition. The third measure was functioning as indexed by the GAF, which was higher in the non-transition than the transition group (*p* < 0.001; see Table 1) and added to the prediction of those individuals who did not make transition.

### 3.2 Prediction of functioning

SVM was able to discriminate between the two subgroups with a specificity of 62.5%, a sensitivity of 62.5% and an accuracy of 62.5%; permutation testing indicated that this was statistically significant (*p* = 0.008). Consistent with this finding, the use of SVR allowed quantitative prediction of functioning with statistically significant accuracy (Person correlation *r* = 0.275, *p* = 0.009; mean squared-error = 376).

**Fig. 2:**
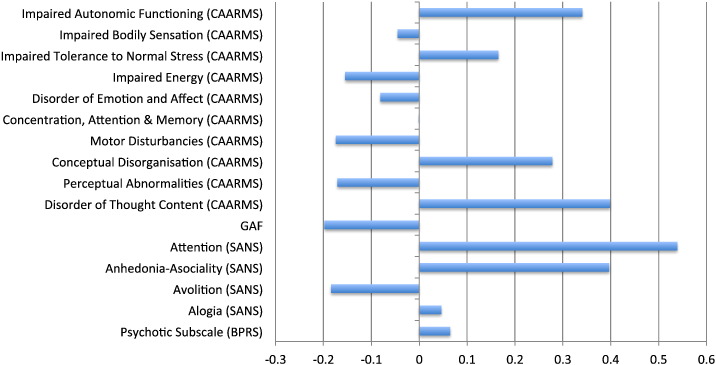
Relative contributions of clinical measures to prediction of long-term functional outcome (i.e. poor versus good functioning). A positive weight value indicates that a measure contains valuable information for identifying low-functioning individuals, whereas a negative value indicates that it contains valuable information for identifying high-functioning individuals.

The relative contributions of the different symptoms to prediction of functional outcome are displayed in Fig. 2. Here a positive weight means that the measure in question contained valuable information for identifying individuals who were low functioning, whereas a negative weight means that it was useful for identifying individuals who were high functioning. It can be seen that individualized predictions were driven by several measures, such as attention disturbances as measured by the SANS, which were more pronounced in the low- than the high-functioning group (*p* < 0.001; see Table 1); anhedonia–asociality as measured by the SANS, which was more pronounced in the low- than the high-functioning group (*p* = 0.009; see Table 1); and disorder of thought content as measured by the CAARMS, which did not differ between high- and low-functioning groups based on group-level statistics (*p* = 0.091; see Table 1). All three measures were associated with positive weight values (see Fig. 2), indicating that they contained valuable information for identifying low-functioning individuals.

## 4 Discussion

Previous studies had shown an association between psychopathology and subsequent clinical or functional outcome in people at UHR for psychosis (Cannon et al., 2008, Nelson et al., 2013, Thompson et al., 2011, Velthorst et al., 2009, Yung et al., 2015, Ziermans et al., 2014). However, effects that are statistically significant at a *group* level do not necessarily permit accurate inferences at the level of the *individual*. The results of the present investigation expands the existing literature by showing that psychopathological measures allow individualized predictions in people at UHR for psychosis. Specifically, we found that a combination of clinical data acquired using the BPRS, SANS, CAARMS and GAF predicted transition to psychosis and functioning with above-chance accuracies of 64.6% and 62.5% respectively. In addition, the use of a parametric approach allowed quantitative prediction of functioning with statistically significant accuracy (*p* = 0.007).

We note that statistically significant accuracy does not necessarily imply clinical utility in real-world clinical practice. The clinical utility of a prognostic test depends on several aspects such as the ability to generate a “divergent prediction” and the availability of alternative interventions (Mechelli et al., 2015, Perlis, 2011). For example, a test that is accurate at predicting a given outcome of interest may not be particularly useful if that outcome is only observed in a very small fraction of the patient population, while a test that is accurate at predicting a highly heterogeneous clinical outcome could be of clinical value. In the context of individuals at UHR for psychosis, the ability to predict transition with an accuracy of 64.6% may be of little clinical value if the risk of transition to psychosis is small (Fusar-Poli et al., 2012, Yung et al., 2007). The eventual development of tools for tailoring intervention to the level of risk in this clinical population, therefore, will ultimately require greater levels of accuracy, sensitivity and specificity than those found in the present investigation.

Recent studies (Addington et al., 2015, Cannon et al., 2008, Cotter et al., 2014, Lencz et al., 2006, Michel et al., 2014, Nieman et al., 2014) suggest that greater levels of accuracy, sensitivity and specificity could be achieved through the integration of psychopathological measures with other types of data. For example, it is possible to refine prediction of transition to psychosis by combined psychopathological information with measures of genetic risk (Thompson et al., 2013), years of education (Ruhrmann et al., 2010), substance abuse (Thompson et al., 2013), sleep disturbances (Ruhrmann et al., 2010), premorbid adjustment (Nieman et al., 2014), cognitive impairment (Lencz et al., 2006, Michel et al., 2014, Riecher-Rossler et al., 2009) and neurophysiology (Nieman et al., 2014). It should be noted, however, that in these studies prediction was typically based on the development of a single cut-off score that was estimated at group rather than individual level. In addition, none of these studies examined the generalizability of the predictive model using a cross-validation procedure that employed separate training and testing data. Future studies could use multivariate supervised machine learning techniques to integrate different types of data, with the aim of improving on the levels of accuracy, sensitivity and specificity that were observed in the present investigation.

Interestingly, individualized prediction of transition to psychosis was mainly driven by disorder of thought content, intensity of attenuated positive symptoms and functioning. This aspect of our results is consistent with previous studies (Addington et al., 2015, Carrion et al., 2013, Nelson et al., 2012, Nelson et al., 2013, Thompson et al., 2013) and emphasizes the importance of considering functioning in the clinical management of people at UHR for psychosis (Cotter et al., 2014). In addition, the observation that disorder of thought content, intensity of attenuated psychotic symptoms and functioning made independent contributions to prediction, suggests that these three aspects reflect independent rather than overlapping processes along the pathway to psychosis. In contrast, long-term functional outcome was mainly informed by attention disturbances, anhedonia–asociality and disorder of thought content. Therefore, functional outcome depends on a diverse collection of features that are overlapping with but distinct from those influencing transition to psychosis.

We note that the levels of accuracy, sensitivity and specificity in the present investigation were lower than in those found in similar studies that have employed neuroimaging data (McGuire et al., 2015). For example, using structural Magnetic Resonance Imaging (MRI) data, Koutsouleris et al. (2015) developed an algorithm that predicted transition to psychosis with an accuracy of 80.4%, a sensitivity of 75.8% and a specificity of 85.0%. In addition, using MRI data, the same research group was able to predict functional outcome with an accuracy of 81.6%, a sensitivity of 78.6% and a specificity of 84.6% (Kambeitz-Ilankovic et al., 2015). A possible explanation is that psychopathological measures are less directly related to the pathophysiological processes that underlie transition to psychosis than neuroimaging data. Another potential explanation is that the present investigation focused on prediction of long-term outcomes (i.e. 7.5 years on average), whereas studies that employed neuroimaging data used much shorter follow-ups (i.e. up to 2 years). Nevertheless, there are several advantages associated with the use of psychopathological measures in everyday psychiatric practice. First, clinical tests are available to psychiatric services in developed as well as developing countries; in contrast, neuroimaging is still only available to a small fraction of the 1.5 million people in the world who develop schizophrenia each year. Second, clinical tests are relatively easy to administer and interpret; while the neuroanatomical alterations associated with transition to psychosis can only be detected after a series of analytical steps that require technical expertise and computational resources beyond the capabilities of most clinical units. Third, most clinical tests can be scored within a short time allowing clinicians to make prompt treatment decisions; in contrast, the statistical analysis of neuroimaging data can take hours or days to complete. It would be impractical and potentially harmful to the patient to delay a treatment decision until the results of such analysis become available. In light of these advantages, psychopathology could be used to inform the clinical management of people at UHR until other techniques such as neuroimaging become widely available.

The present investigation has several strengths. In particular, (i) the application of SVM to the data allowed us to make statistical inferences at the level of the individual rather than the group; (ii) the sample size was considerably larger than in any previous study using supervised machine learning techniques to predict outcomes in the UHR population; (iii) participants in different sub-groups (i.e. transition versus non-transition; poor versus good functioning) were individually matched for age and gender; and (iv) outcome was assessed not only in terms of transition to psychosis but also in terms of functioning. The present investigation also has important limitations. Firstly, we focused on the predictive value of clinical and functional information without considering other types of data that might improve prognostic accuracy. Secondly, most participants received psychosocial and/or pharmacological treatment over the follow-up period; this raises the possibility that our findings might reflect individual differences in response to treatment rather than putative prognostic risk. We note that this limitation is not specific to the present investigation but applies to most, if not all, studies of people at UHR for psychosis. Thirdly, there were minor modifications to the UHR criteria and instruments used to assess these over the recruitment period although there is no reason to expect that this had significant effects (Nelson et al., 2013). Fourthly, the high degree of variability in follow-up time (see Materials and methods for detail) may have introduced noise to the data, resulting in an under-estimation of predictive accuracies.

In conclusion, our findings demonstrate that psychopathological features allow individualized prognostic predictions in people at UHR for psychosis with statistically significant accuracy. However, we argue that the eventual development of prognostic tools for predicting risk and tailoring intervention in this clinical population, will ultimately require greater levels of accuracy, sensitivity and specificity than those reported in the present study. This could be achieved by combining psychopathological information with other types of data using a multivariate supervised machine learning framework (Pettersson-Yeo et al., 2014).

## Role of funding source

No funding body agreements. See list of funders under acknowledgements.

## Conflict of interest

None.

## Contributors

Andrea Mechelli, Stephen Wood, Patrick McGorry, Paul Amminger, Barnaby Nelson and Alison Yung designed the research project. Ashleigh Lin, Stephen Wood and Paul Amminger acquired the data; Andrea Mechelli, Stefania Tognin and Jonathan Young analysed the data; Andrea Mechelli, Philip McGuire, Barnaby Nelson and Alison Yung wrote the paper. All authors reviewed the manuscript.

## Appendix A Supplementary data

Supplementary material. Image 1
