# Supplementary material for: Using clinical information to make individualized prognostic predictions in people at ultra high risk for psychosis
Source: Schizophr Res. 2017 Jun;184:32–8. doi: 10.1016/j.schres.2016.11.047 (PMC5477095; doi:10.1016/j.schres.2016.11.047)
Supplement: Supplementary file 1 — Supplementary material. [file mmc1.docx]

**SUPPLEMENTARY DATA**

**Using Clinical Information to Make Individualized Prognostic Predictions in People at Ultra High Risk for Psychosis**

Andrea Mechelli^1^, Ashleigh Lin^2^, Stephen Wood^3,4^, Patrick McGorry^5,6^,

Paul Amminger^5,6^, Stefania Tognin^1^, Philip McGuire^1^, Jonathan Young^7^,

Barnaby Nelson^5,6^*, Alison Yung^8,9^*

1. Department of Psychosis Studies, Institute of Psychiatry, Psychology & Neuroscience, King's College London, London, UK.

2. Telethon Kids Institute, University of Western Australia, Subiaco Western Australia 6008

3. Department of Psychology, University of Birmingham, Birmingham, UK.

4. Melbourne Neuropsychiatry Centre, Department of Psychiatry, University of Melbourne & Melbourne Health, Melbourne, Australia

5. Orygen, The National Centre of Excellence in Youth Mental Health, Melbourne, Melbourne, Australia.

6. The Centre for Youth Mental Health, The University of Melbourne, Melbourne, Australia.

7. Department of Neuroimaging, Institute of Psychiatry, Psychology & Neuroscience, King's College London, London, UK.

8. Institute of Brain, Behaviour, and Mental Health, University of Manchester, Manchester, UK.

9. Greater Manchester West NHS Mental Health Foundation Trust, Prestwich, Manchester.

* these two authors contributed to this work equally

*Baseline Measures.* The following clinical measures acquired at baseline were used to predict clinical and functional outcomes: the psychotic subscale of the Brief Psychiatric Rating Scale (BPRS); four subscales from the Scale for Assessment of Negative Symptoms, (SANS) (i.e. “Alogia”, “Avolition”, “Anhedonia”, “Attention”); ten subscales from the Comprehensive Assessment of At Risk Mental State (CAARMS) (i.e. “Disorder of Thought Content”, “Perceptual Abnormalities”, “Conceptual Disorganization”, “Motor Disturbances”, “Disorders of Concentration, Attention and Memory”, “Disorders of Emotion and Affect”, “Impaired Energy”, “Impaired Tolerance to Normal Stress”, “Impaired Bodily Sensations” and “Impaired Autonomic Functioning”); and the Global Assessment of Functioning (GAF).
